# Supplementary material for: Extreme slow growth as alternative strategy to survive deep starvation in bacteria
Source: Nat Commun. 2019 Feb 21;10:890. doi: 10.1038/s41467-019-08719-8 (PMC6385201; doi:10.1038/s41467-019-08719-8)
Supplement: Supplementary file 1 — Supplementary Information [file 41467_2019_8719_MOESM1_ESM.pdf]

Supplementary Information

Extreme slow growth as alternative strategy to survive deep starvation in bacteria

Gray *et al.*

- Supplementary Table 1 Strains and plasmids used in this study
- Supplementary Table 2 Primers used to confirm clean deletions
- Supplementary Figure 1 Deep starvation assay
- Supplementary Figure 2 Spores and cells in wild type *B. subtilis* cultures
- Supplementary Figure 3 Survival of a *B. subtilis* germination mutant
- Supplementary Figure 4 Membrane potential of starved cells
- Supplementary Figure 5 GFP expression capacity during deep starvation
- Supplementary Figure 6 Nascent protein and peptidoglycan synthesis
- Supplementary Figure 7 Effect of 3-MBA on wild type cells and a resistant mutant
- Supplementary Figure 8 Protein concentration in starvation buffer
- Supplementary Figure 9 Survival of different mutants
- Supplementary References

Supplementary Table 1. Strains and plasmids used in this study

Strain	Genotype	Construction, source or reference
BSB1	*trp+*	Lab strain
1803	*divIVA::(pdivIVA-gfp divIVA::chl)*	[1]
4626	*trpC2, Ω(spoIIE::erm) amyE::(spc Pxyl-gfpmut1-yqgS)*	[2]
PG344	*spoIIE::erm*	P. Gamba, unpublished
PG344_2	*spoIIE::er(spec)m*	This work
DG001	*spoIIE::erm, divIVA::(pdivIVA-gfp divIVA::chl)*	This work
JWV026	*spo0A::kan*	J.-W. Veening, unpublished
DG004	*spoIIE::erm, divIVA::(pdivIVA-gfp divIVA::chl), spo0A::kan*	This work
BUG1	*clpP::spec*	[3]
DG005	*spoIIE::erm, divIVA::(pdivIVA-gfp divIVA::chl), clpP::spec*	This work
WB800	*nprE aprE epr bpr mpr::ble nprB::bsr vpr wprA::hyg*	[4]
ΔWB800	*nprE aprE epr bpr mpr::ble nprB::bsr vpr wprA::hyg spoIIE::erm*	This work
Δ6 phage	*trpC2, ΔSPb, sublancin 168-sensitive, Δskin, ΔPBSX, Δprophage1, pks::chl, Δprophage 3::chl*	[5]
Δphage	*trpC2, ΔSPb, sublancin 168-sensitive, Δskin, ΔPBSX, Δprophage1, pks::chl, Δprophage 3::chl, spoIIE::erm*	This work
2682	*trpC2 recA::tet*	L.J. Wu
DG010	*spoIIE::erm, divIVA::(pdivIVA-gfp divIVA::chl), recA::tet*	This work
BRB01	*trpC2, nprB*	[6]
BRB02	*trpC2, nprB, aprE*	[6]
BRB03	*trpC2, nprB, aprE, epr*	[6]
BRB04	*trpC2, nprB, aprE, epr, bpr*	[6]
BRB05	*trpC2, nprB, aprE, epr, bpr, nprE,*	[6]
BRB06	*trpC2, nprB, aprE, epr, bpr, nprE, mpr*	[6]
BRB07	*trpC2, nprB, aprE, epr, bpr, nprE, mpr, vpr*	[6]
BRB08	*trpC2, nprB, aprE, epr, bpr, nprE, mpr, vpr, wprA*	[6]
Δ1 protease	*trpC2, nprB, spoIIE::erm*	This work
Δ2 protease	*trpC2, nprB, aprE, spoIIE::erm*	This work
Δ3 protease	*trpC2, nprB, aprE, epr, spoIIE::erm*	This work
Δ4 protease	*trpC2, nprB, aprE, epr, bpr, spoIIE::erm*	This work
Δ5 protease	*trpC2, nprB, aprE, epr, bpr, nprE, spoIIE::erm*	This work
Δ6 protease	*trpC2, nprB, aprE, epr, bpr, nprE, mpr, spoIIE::erm*	This work
Δ7 protease	*trpC2, nprB, aprE, epr, bpr, nprE, mpr, vpr, spoIIE::erm*	This work
Δ8 protease	*trpC2, nprB, aprE, epr, bpr, nprE, mpr, vpr, wprA, spoIIE::erm*	This work
BKE10770	*trpC2, wprA::erm*	BGSC
DG034	*wprA, spoIIE::erm*	This work

BKE24160	*trpC2, mmgB::erm*	BGSC
DG097	*spoIIE::er(spec)m, mmgB::erm*	This work
BKE12020	*trpC2, manA::erm*	BGSC
DG100	*spoIIE::er(spec)m, manA::erm*	This work
BKE08060	*trpC2, acoA::erm*	BGSC
DG091	*spoIIE::er(spec)m, acoA::erm*	This work
BKE10740	*trpC2, yisJ::erm*	BGSC
DG095	*spoIIE::er(spec)m, yisJ::erm*	This work
BKE07190	*trpC2, yezD::erm*	BGSC
DG092	*spoIIE::er(spec)m, yezD::erm*	This work
OC003	*abrB::spec*	[7]
DG024	*spoIIE::erm, abrB::spec*	This work
GP959	*trpC2, sinI::spec*	[8]
DG019	*spoIIE::erm, sinI::spec*	This work
codY	*codY::spec*	S. Syvertsson
DG051	*spoIIE::erm, divIV-gfp::chl, codY::spec*	This work
8G32	*comK::kan*	[9]
DG021	*spoIIE::erm, divIV-gfp::chl, comK::kan*	This work
Δ*sigD*	*sigD::kan*	S. Syvertsson, unpublished
DG049	*spoIIE:erm, divIV-gfp::chl, sigD::kan*	This work
QB5344	*sigB::chl*	J. Stülke
DG057	*spoIIE::erm, sigB::chl*	This work
BG546	*BG1, pnpA::kan*	[10]
DG048	*spoIIE:erm, divIV-gfp::chl, pnpA::kan*	This work
BKE25750	*trpC2, nucB::erm*	BGSC
DG067	*spoIIE::er(spec)m, nucB::erm*	This work
BD2941	*nucA::spec*	[11]
DG047	*spoIIE::erm, nucA::spec*	This work
1A792	*trpC2, lytABC::neo, lytD::tet, lytE::chl, lytF::spec*	[12]
DG022	*lytABC::neo, spoIIE::erm*	This work
BKE27600	*relA::erm*	BGSC
DG077	*spoIIE::er(spec)m, relA::erm*	This work
1A905	*sigW::erm*	BGSC
DG129	*spoIIE::er(spec)m, sigW::erm*	This work
HB10216	*sigM::kan*	[13]
DG132	*spoIIE::erm, sigM::kan*	This work
GP146	*sigL::spec*	[14]
DG131	*spoIIE::erm, sigL::spec*	This work
HB7007	*sigX::spec*	[15]
DG123	*spoIIE::erm, sigX::spec*	This work
Δ*sigG*	*trpC2, (spoIIIG::ermC)731*	J. Errington lab stocks
DG128	*spoIIE::er(spec)m, (spoIIIG::ermC)731*	This work
4265	*sigI, rsgI::neo*	[16]
DG133	*sigI, rsgI::neo, spoIIE::erm*	This work
BKE27120	*sigV::erm*	BGSC
DG126	*spoIIE::er(spec)m, sigV::erm*	This work
ΔsigK	*sigK::erm*	BGSC
DG124	*spoIIE::er(spec)m, sigK::erm*	This work
Δ*sigF*	*trpC2, (spoIIAC::chl)678*	J. Errington lab stocks
DG130	*spoIIE::erm, (spoIIAC::chl)678*	This work
BKE27120	*trpC2, sigE::erm*	BGSC
DG125	*spoIIE::er(spec)m, sigE::erm*	This work
BKE35500	*trpC2, degS::ery*	BGSC

DG122	*spoIIE::er(spec)m, degS::ery*	This work
BD1818	*degU::chl*	PHRI*
DG016	*spoIIE::erm, degU::chl*	This work
BKE01700	*trpC2, murQ::erm*	BGSC
DG121	*spoIIE::er(spec)m, murQ::erm*	This work
DG016	*spoIIE::erm, degU::chl*	This work
TE47	*amyE::(Phyperspank-sfGFP*-ilvD(terminator) lacI(cis) spc)*	T. Ewen, unpublished
DG017	*amyE::(Phyperspank-sfGFP*-ilvD(terminator) lacI(cis) spc), spoIIE::erm*	This work
BKE00980	*sigH::erm*	BGSC
BWW1	*spoIIE::er(spec)m, sigH::erm*	This work
FB113	*cwlJ::tet sleB::spc*	[17]
DWA27	*ftsZ*-G196S-N263K (*cat*)	[18]
HS545	*ftsZ*-G196S-N263K (*cat*), *spoIIE::erm*	This work
KS15	*dacA::spc*	K. Rafiq & R. Daniel, thesis K. Rafiq
**Plasmid**	**Genotype**	**Source or reference**
pDR244	*cre, spec, amp*	BGSC
pErm::Spec	*erm::spec*	[19]

*Public Health Research Institute Centre

Supplementary Table 2. Primers used to confirm clean deletions

Primer	Sequence	Gene
DG1	5′-GATCCTCCGGTGCTTGTG-3′	*Forward aprE*
DG2	5′-GGCCGCATCTGATGTCTTTG-3′	*Reverse aprE*
DG3	5′-GATACGCTTGACATCCCGAC-3′	*Forward bpr*
DG4	5′-GAACGCTCCGCCTACCAG-3′	*Reverse bpr*
DG5	5′-GCGCGATCCTTCACATAGCC-3′	*Forward nprE*
DG6	5′-GCCTCATTCGGTTAGACAGCG-3′	*Reverse nprE*
DG7	5′-CACCCGAGTGAATGTGC-3′	*Forward epr*
DG8	5′-CCTGCGAGCAGCAGTAATTC-3′	*Reverse epr*
DG9	5′-GCGGATTACACTGTTGAAGG-3′	*Forward mpr*
DG10	5′-CTCTGTACTCGGCTCCTCATC-3′	*Reverse mpr*
DG11	5′-GCTTATACTGGCATATGGAGC-3′	*Forward nprB*
DG12	5′-CATCGAGCTTATGAAAGAGCG-3′	*Reverse nprB*
DG13	5′-CTTAATCACAAGAGATATCCAC-3′	*Forward vpr*
DG14	5′-CTTATGAACAGAGACGAATTGC-3′	*Reverse vpr*
DG15	5′-GGAGGCCTGTGGGTCGGCTTC-3′	*Forward wprA*
DG16	5′-CGGCTTATCGGTATTCGATTGC-3′	*Reverse wprA*
DG17	5′-TCGCGACATAGCGGTTGTTTCTGAGC-3′	*Forward sigI*
DG18	5′-GAGCATAATAGCAGTCATCG-3′	*Reverse sigI*

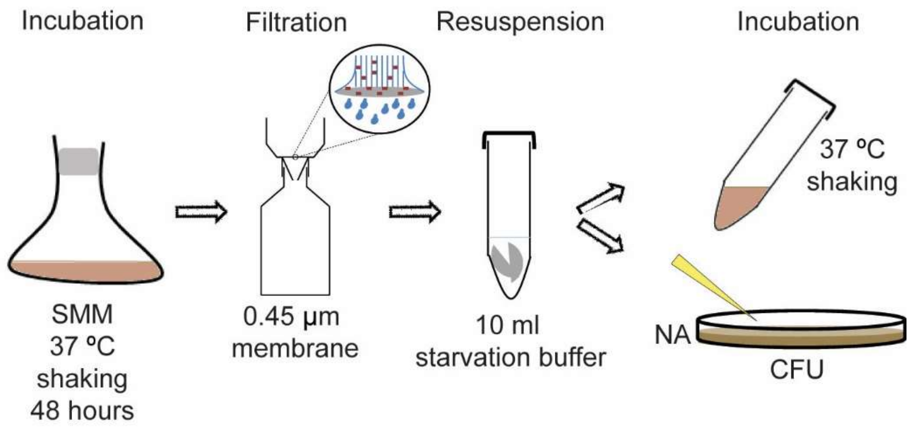

Supplementary Figure 1. Deep starvation assay

Cartoon of the deep starvation assay. Strains were cultured in 10 ml SMM at 37ºC with shaking for 48 hours followed by filtration using 47 mm filter membranes with 0.45 µm pores size (ThermoFisher). Cells were subsequently resuspended in 10 ml starvation buffer (15 mM $(NH_4)_2SO4$, 80 mM K_2HPO_4, 44 mM KH_2PO_4, 50 mM NaCl, 0.8 mM $MgSO_4$), and incubated at 37ºC under continuous shaking for 14 days. Periodic sampling was performed to determine the CFU through serial dilutions and plating on nutrient agar.

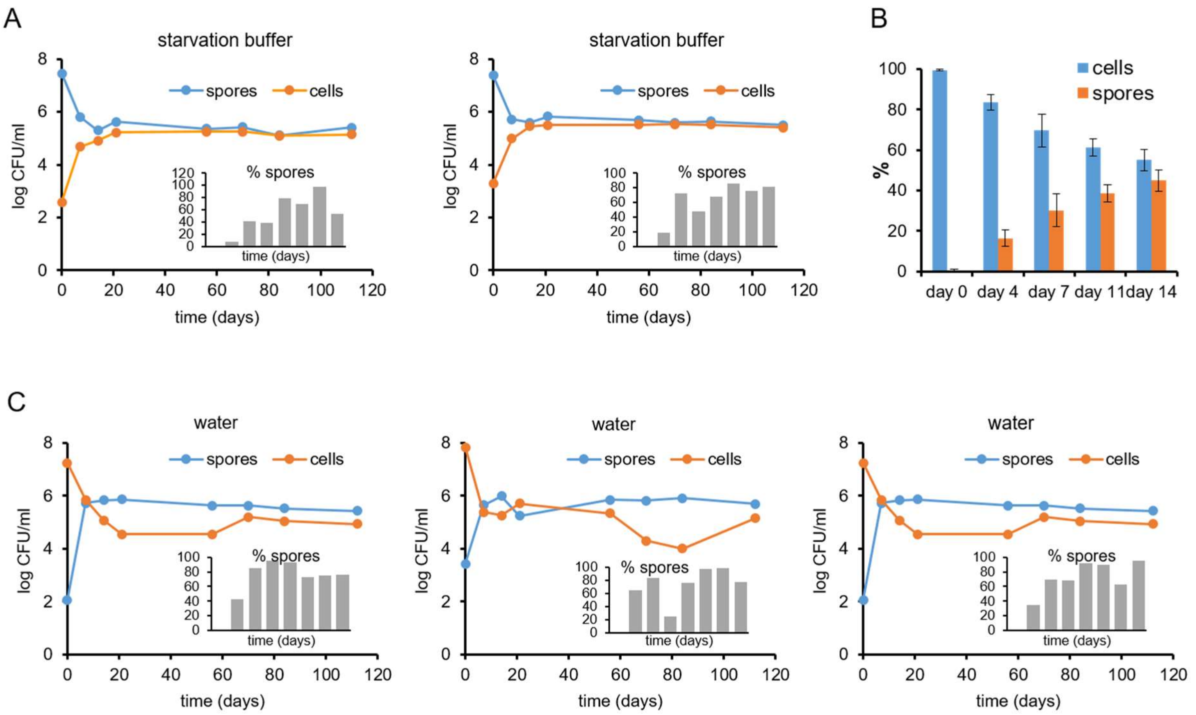

Supplementary Figure 2. Spores and cells in wild type *B. subtilis* cultures

(A) CFU of spores and cells in a wild type *B. subtilis* culture (strain BSB1) incubated in starvation buffer. The percentage of spores is indicated by the bar diagram. These are the biological replicates of Fig. 1C in the main text. (B) Fraction of spores and non-sporulating cells measured using phase-contrast microscopy. Results from three biological replicates are shown. (C) Three biological replicates of CFU of spores and cells in a wild type *B. subtilis* culture (strain BSB1) incubated in water. The percentage of spores is indicated by the bar diagram.

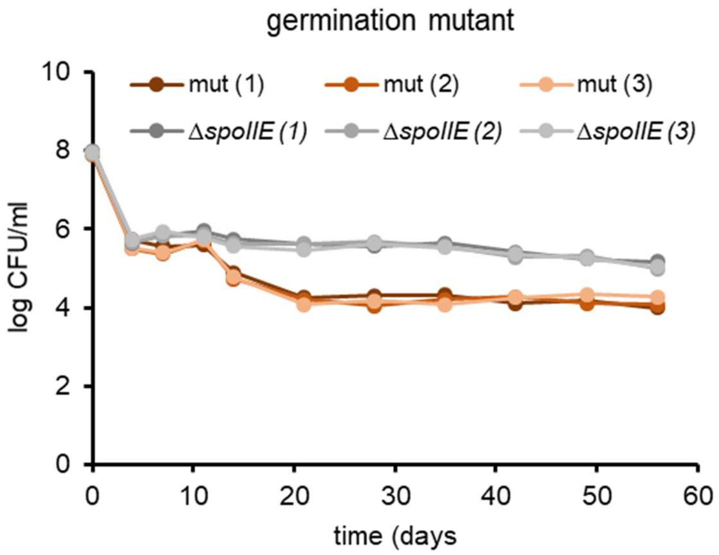

Supplementary Figure 3. Survival of a *B. subtilis* germination mutant

CFU of *B. subtilis* germination mutant (mut) FB113 (Δ*cwlJ* Δ*sleB*)[17] and the Δ*spoIIE* control. Both strains were incubated in starvation buffer. Three biological replicates are shown.

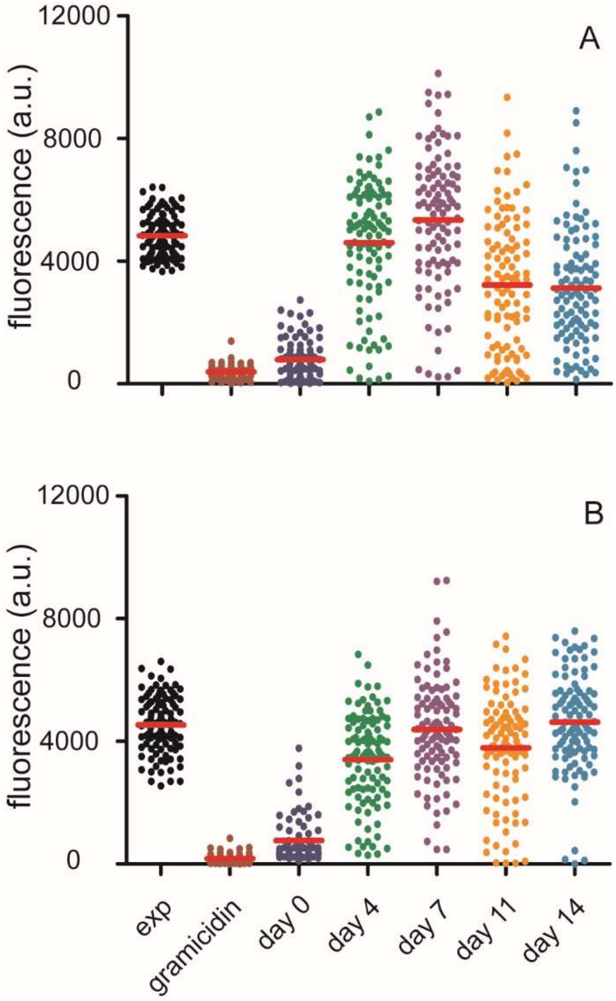

Supplementary Figure 4. Membrane potential levels of starved cells

(A & B) Two biological replicates of Fig. 3E in the main text. After 7, 11 and 14 days deep starvation the relative membrane potential was measured in individual cells by following the uptake of the membrane potential sensitive fluorescence dye $DiSC_3(5)$. As controls, exponentially growing cells (OD_{600} 0.2), and cells treated with 10 µg/ml gramicidin ABC were determined. Fluorescence intensities of approximately 100 cells were quantified.

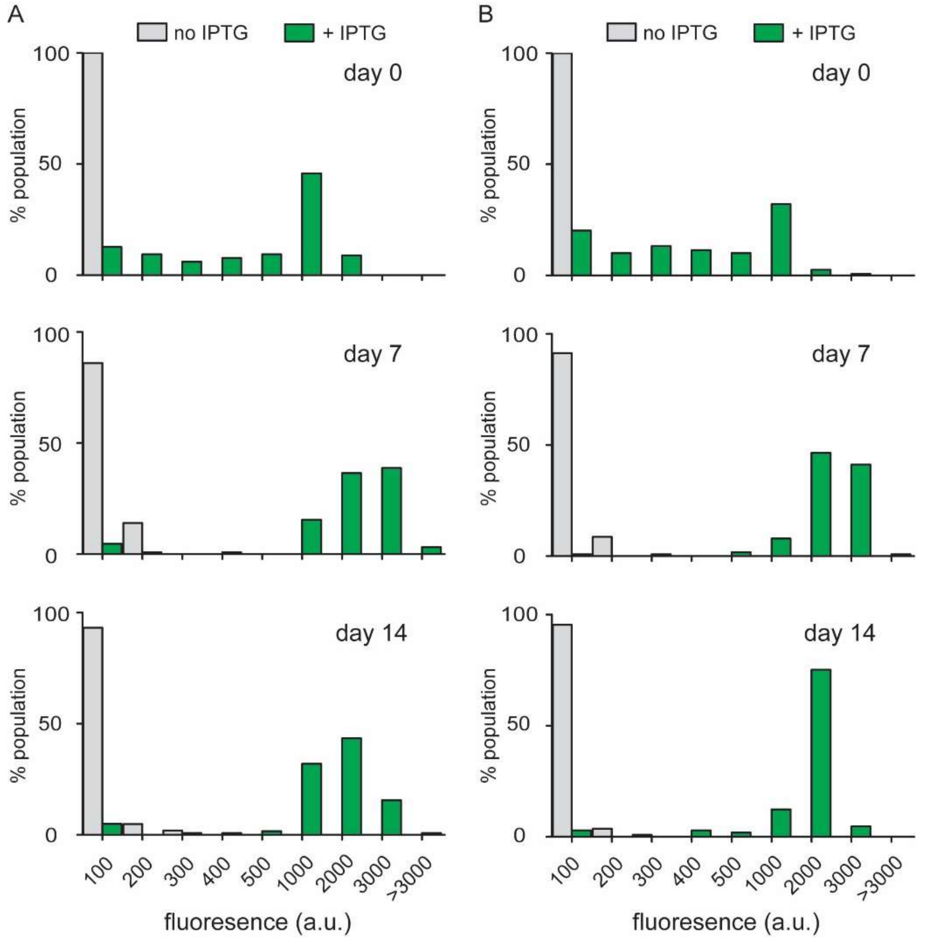

Supplementary Figure 5. GFP expression capacity during deep starvation (A & B) Two biological replicates of Fig. 4 in the main text. *B. subtilis* strain Δ*spoIIE* containing an inducible GFP reporter (*amyE::Phyperspank-sfGFP*) was incubated in starvation buffer for 0, 7 and 14 days, followed by incubation in the presence and absence of 1 mM IPTG for 4 hours. The fluorescence of approximately 100 cells was quantified.

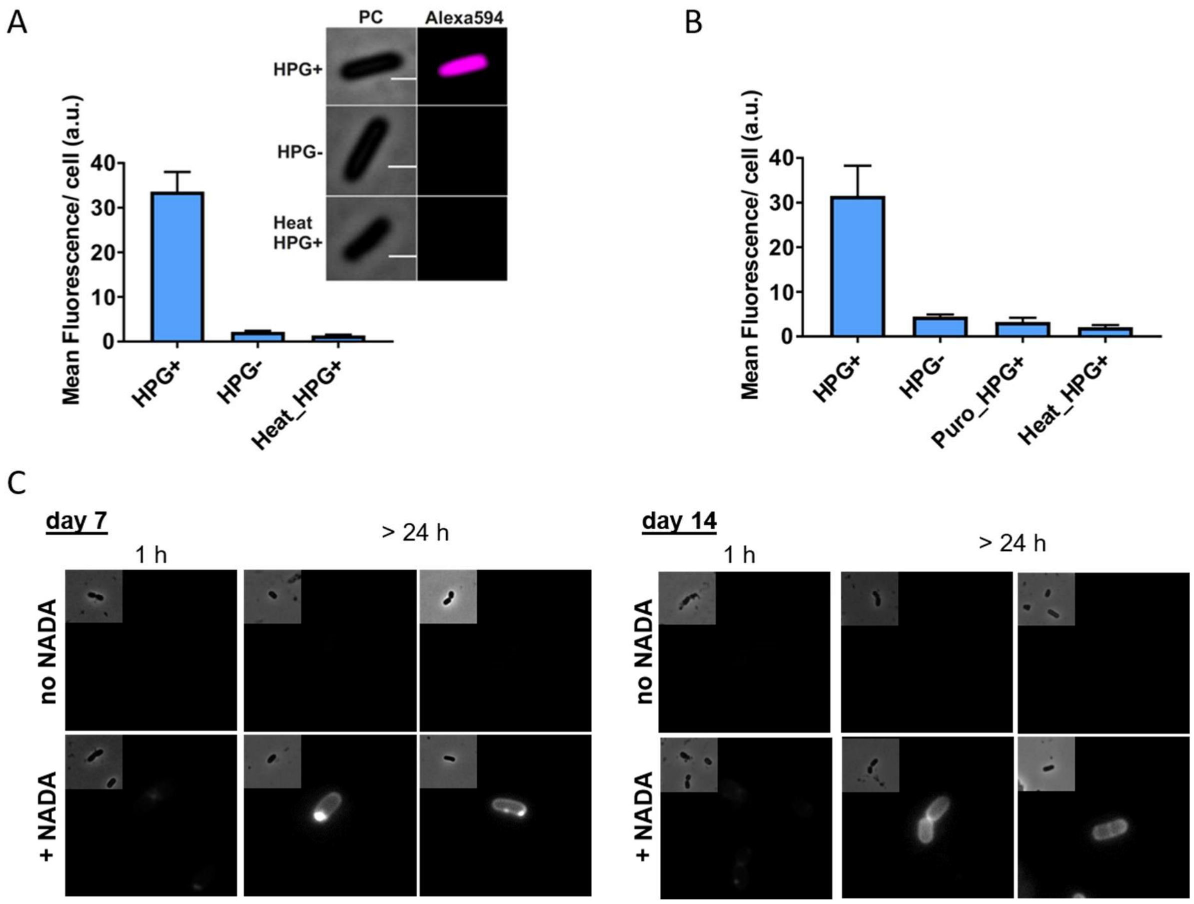

Supplementary Figure 6. Nascent protein and peptidoglycan synthesis

B. subtilis Δ*spoIIE* cells (strain DG001) were cultured for 14 days (A) or 19 days (B) in starvation buffer and incubated with the amino acid analog L-homopropargylglycine (HPG). Incorporated HPG was fluorescently labelled with Alexa594-azide using click chemistry[20]. Inset in (A) shows a representative microscopic image (bar 1 μm). As controls cells were heat-killed (Heat_HPG+) before incubation with HPG or incubated with the translation inhibitor puromycin (Puro_HPG+). Bar diagrams show mean Alexa594 fluorescence (± SEM) in cells (60 and 45 cells for (A) and (B), respectively). (C) Peptidoglycan labelling with 5 μM NADA[21]. Cells were incubated with NADA for 48 hrs and resuspended in Starvation buffer. Samples were taken after 1 h and > 24 h (two examples shown).

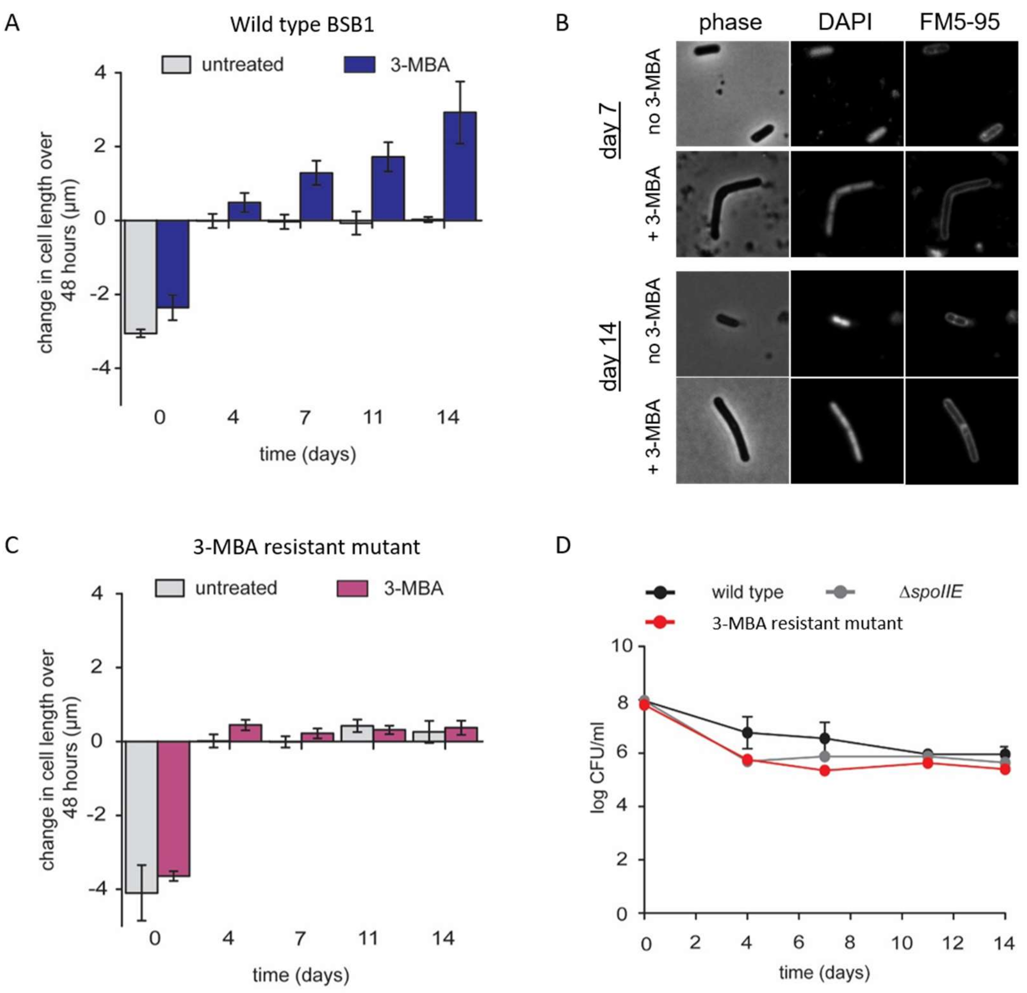

Supplementary Figure 7. Effect of 3-MBA on wild type cells and on a resistant mutant

(A) *B. subtilis* wild type strain BSB1, was incubated for 14 days in starvation buffer. At regular time intervals samples were withdrawn and incubated with 3-MBA for 48 hours, and the average change in cell length was calculated for approximately 100 individual cells for each time point. Bar diagrams depict the average and standard deviation of 3 independent experiments. (B) Representative picture of DNA (DAPI) and membrane (FM5-95) stained 7 and 17 days starved Δ*spoIIE* cells (strain DG001) treated without or with 3-BMA (48 h). (C) *B. subtilis* mutant DWA27, which contains two FtsZ mutations (G196S, N263K) rendering the strain resistant to the cell division inhibitor 3-methoxybenzamide (3-MBA)[18], treated with 3-BMA as described in (A). (D) Average CFUs of the related cultures.

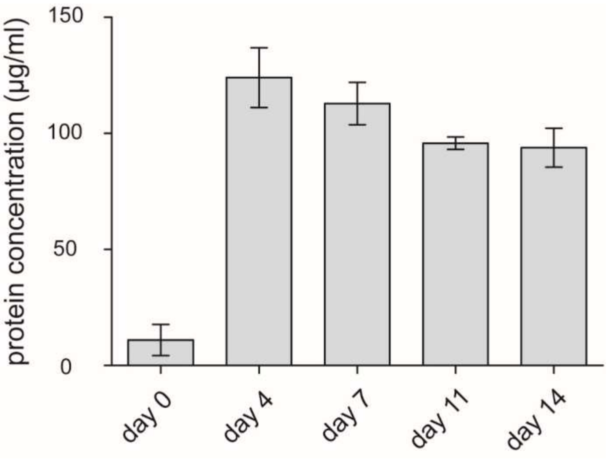

Supplementary Figure 8. Protein concentration in starvation buffer

Concentration of proteins released in the medium by lysed cells during deep starvation of *B. subtilis* Δ*spoIIE* cells as measured using a Bradford assay. Bar diagram represents average and standard deviation of 3 independent experiments.

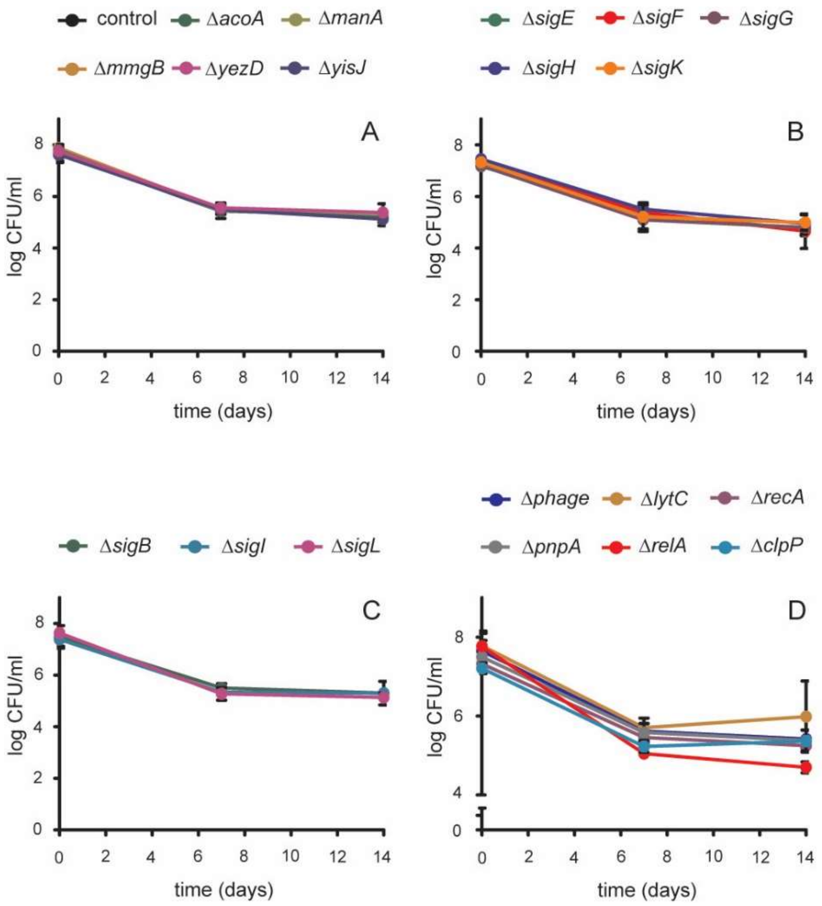

Supplementary Figure 9. Survival of different mutants

(A) Survival of mutants that were selected from the up-regulated genes under deep starvation conditions. (B) Survival of cells lacking sporulation sigma factors (Δ*sigE*, -*F*, -*G*, -*H*, -*K*). (C) Survival of cells lacking either the general stress response sigma factor SigB, the cold shock sigma factor SigL or the heat shock sigma factors SigI. (D) Survival of cells lacking either the major autolysin LytC, the protein quality control protease ClpP, the stringent response regulator RelA, the DNA recombination/repair proteins RecA or PnpA (only log 4-8 CFU/ml is shown to emphasize differences). Graphs represent averages and standard deviations of 3 independent experiments. The Δ*spoIIE* background strain was used in all experiments.

Supplementary References

1 Thomaides, H. B., Freeman, M., El Karoui, M. & Errington, J. Division site selection protein DivIVA of Bacillus subtilis has a second distinct function in chromosome segregation during sporulation. *Genes Dev.* **15**, 1662-1673 (2001).

2 Schirner, K., Marles-Wright, J., Lewis, R. J. & Errington, J. Distinct and essential morphogenic functions for wall- and lipo-teichoic acids in Bacillus subtilis. *EMBO J.* **28**, 830-842 (2009).

3 Gerth, U. *et al.* Fine-tuning in regulation of Clp protein content in Bacillus subtilis. *J. Bacteriol.* **186**, 179-191 (2004).

4 Wu, S. C. *et al.* Functional production and characterization of a fibrin-specific single-chain antibody fragment from *Bacillus subtilis*: effects of molecular chaperones and a wall-bound protease on antibody fragment production. *Appl. Environ. Microbiol.* **68**, 3261-3269 (2002).

5 Westers, H. *et al.* Genome engineering reveals large dispensable regions in *Bacillus subtilis*. *Mol Biol Evol* **20**, 2076-2090 (2003).

6 Pohl, S. *et al.* Proteomic analysis of *Bacillus subtilis* strains engineered for improved production of heterologous proteins. *Proteomics* **13**, 3298-3308 (2013).

7 Chumsakul, O. *et al.* Genome-wide binding profiles of the *Bacillus subtilis* transition state regulator AbrB and its homolog Abh reveals their interactive role in transcriptional regulation. *Nucleic Acids Res.* **39**, 414-428 (2011).

8 Diethmaier, C. *et al.* A novel factor controlling bistability in Bacillus subtilis: the YmdB protein affects flagellin expression and biofilm formation. *J. Bacteriol.* **193**, 5997-6007 (2011).

9 Smits, W. K. *et al.* Temporal separation of distinct differentiation pathways by a dual specificity Rap-Phr system in Bacillus subtilis. *Mol Microbiol* **65**, 103-120 (2007).

10 Liu, B. *et al.* Global analysis of mRNA decay intermediates in Bacillus subtilis wild-type and polynucleotide phosphorylase-deletion strains. *Mol. Microbiol.* **94**, 41-55 (2014).

11 Provvedi, R., Chen, I. & Dubnau, D. NucA is required for DNA cleavage during transformation of Bacillus subtilis. *Mol. Microbiol.* **40**, 634-644 (2001).

12 Margot, P., Pagni, M. & Karamata, D. Bacillus subtilis 168 gene lytF encodes a gamma-D-glutamate-meso-diaminopimelate muropeptidase expressed by the alternative vegetative sigma factor, sigmaD. *Microbiology* **145 (Pt 1)**, 57-65 (1999).

13 Luo, Y. & Helmann, J. D. Analysis of the role of Bacillus subtilis sigma(M) in beta-lactam resistance reveals an essential role for c-di-AMP in peptidoglycan homeostasis. *Mol. Microbiol.* **83**, 623-639 (2012).

14 Commichau, F. M. *et al.* Characterization of Bacillus subtilis mutants with carbon source-independent glutamate biosynthesis. *Journal of molecular microbiology and biotechnology* **12**, 106-113 (2007).

15 Huang, X. J., Decatur, A., Sorokin, A. & Helmann, J. D. The Bacillus subtilis sigma(X) protein is an extracytoplasmic function sigma factor contributing to survival at high temperature. *J. Bacteriol.* **179**, 2915-2921 (1997).

16 Schirner, K. & Errington, J. The cell wall regulator {sigma}I specifically suppresses the lethal phenotype of mbl mutants in *Bacillus subtilis*. *J. Bacteriol.* **191**, 1404-1413 (2009).

17 Paidhungat, M. & Setlow, P. Role of ger proteins in nutrient and nonnutrient triggering of spore germination in *Bacillus subtilis*. *J. Bacteriol.* **182**, 2513-2519. (2000).

18 Adams, D. W., Wu, L. J. & Errington, J. A benzamide-dependent ftsZ mutant reveals residues crucial for Z-ring assembly. *Mol Microbiol* **99**, 1028-1042 (2016).

19 Steinmetz, M. & Richter, R. Plasmids designed to alter the antibiotic resistance expressed by insertion mutations in *Bacillus subtilis,* through *in vivo* recombination. *Gene* **142**, 79-83 (1994).

20 Stempler, O. *et al.* Interspecies nutrient extraction and toxin delivery between bacteria. *Nat Commun* **8**, 315 (2017).

21 Kuru, E. *et al.* In Situ probing of newly synthesized peptidoglycan in live bacteria with fluorescent D-amino acids. *Angewandte Chemie* **51**, 12519-12523 (2012).
